# Reconciling public health common good and individual privacy: new methods and issues in geoprivacy

**DOI:** 10.1186/s12942-022-00300-9

**Published:** 2022-01-19

**Authors:** Maged N. Kamel Boulos, Mei-Po Kwan, Khaled El Emam, Ada Lai-Ling Chung, Song Gao, Douglas B. Richardson

**Affiliations:** 1 Institute for Preventive Medicine and Public Health, School of Medicine (FMUL), University of Lisbon, 1649-028 Lisbon, Portugal; 2 Institute of Space and Earth Information Science, The Chinese University of Hong Kong, Shatin, Hong Kong, China; 3 School of Epidemiology and Public Health, University of Ottawa, Ottawa, ON K1G 5Z3 Canada; 4 Office of the Privacy Commissioner for Personal Data, Wanchai, Hong Kong, China; 5 Department of Geography, University of Wisconsin-Madison, Madison, WI 53706 USA; 6 Centre for Geographic Analysis, Institute for Quantitative Social Science, Harvard University, Cambridge, MA 02138 USA

**Keywords:** Geoprivacy, Location privacy, Privacy enhancing technology, Synthetic data, Machine learning, Public health

## Abstract

This article provides a state-of-the-art summary of location privacy issues and geoprivacy-preserving methods in public health interventions and health research involving disaggregate geographic data about individuals. Synthetic data generation (from real data using machine learning) is discussed in detail as a promising privacy-preserving approach. To fully achieve their goals, privacy-preserving methods should form part of a wider comprehensive socio-technical framework for the appropriate disclosure, use and dissemination of data containing personal identifiable information. Select highlights are also presented from a related December 2021 AAG (American Association of Geographers) webinar that explored ethical and other issues surrounding the use of geospatial data to address public health issues during challenging crises, such as the COVID-19 pandemic.

## Introduction

In 2009, Kamel Boulos et al. published an overview of privacy issues and privacy-preserving methods in public health interventions and health research involving disaggregate geographic data about individuals [1]. Since then, there has been an exponential increase in spatial data about individuals from embedded sensors and cameras, wearables, smartphones and user-generated content on social media. Moreover, new privacy-preserving methods have been introduced, e.g., (quasi) synthetic data generated from real data using machine learning and Apple-Google's privacy-preserving, decentralised smartphone Bluetooth proximity sensing method widely used in digital contact tracing apps during the COVID-19 pandemic.

This article provides an updated state-of-the-art summary of privacy-preserving methods and associated issues, with a special focus on synthetic data generation. Select highlights are also presented from a related December 2021 AAG (American Association of Geographers) webinar that explored ethical and other issues surrounding the use of geospatial data to address public health issues.

### Essential definitions

In micro-scale geographical analyses involving health/care data about specific individuals, data security, confidentiality and privacy form an intertwined triad. Privacy is the individual’s right to control the acquisition, use and disclosure of their identifiable health information, including their geo-tagged information and place history. Confidentiality involves the privacy interests that arise from specific relationships (e.g., doctor/patient, researcher/subject) and corresponding legal and ethical duties. Security covers the technological or administrative safeguards or tools to protect identifiable health information from unwarranted access, use, or disclosure [1].

### Why do we need to perform micro-scale analyses? Why not just use data aggregated to administrative regions?

In 1854, John Snow did his famous spatial analysis exercise to discover and prove the faecal-oral mode of transmission of cholera, and to trace and stop the source of a cholera outbreak in Soho, London, using only manually-collected data (of cases and water pumps) and a hand-drawn map for data visualisation and exploration. This famous map only solved the problem because the unique locations of individual cases were known [2]. Many important clinical and health research studies, as well as public health interventions (e.g., during the COVID-19 pandemic) would not be possible without access to disaggregate geographic data about individuals.

## Privacy and confidentiality-preserving solutions for geolocation

A clash or tension clearly exists between the need to conduct micro-scale analyses (for the common good), such as the one conducted by John Snow in 1854, on the one hand, and individual privacy, including location privacy, on the other. A number of statistical and epidemiological data processing methods (data aggregation and transformations) have been proposed that can be applied to original location data to preserve individuals' privacy while maintaining some acceptable level of data usefulness for geographical analyses. However, the use of precise addresses will continue to be needed in many cases to improve data analysis results or make them possible at all [1].

One example of such transformations is MPT (Multidimensional Point Transform) proposed by AbdelMalik and Kamel Boulos in 2011/2012. MPT integrates the spatial dimension with other dimensions of interest to comprehensively anonymise data and produces a more appropriate transform that builds location privacy into the anonymisation model from the beginning [3].

More recently, other researchers proposed using (quasi) synthetic data in micro-scale analyses. The process involves generating synthetic data from real data using a machine learning model that captures the patterns in real data and then generates new data from that model. The generated non-identifiable data closely match the statistical properties and patterns in the original dataset, offering very similar results and leading to the same conclusions, all while preserving individuals’ privacy and without the legislative need for additional consent [4, 5]. This method is further discussed in detail in the next section.

However, in all of the above methods, there is always this implicit trade-off between privacy concerns (e.g., easiness of re-identification) and the types and accuracy of the results of geographical health analyses that are possible with a given data set (original, unaltered vs. transformed or aggregated data). And that is where software agents can offer a potential solution that preserves the full fidelity of the original data, as proposed by Kamel Boulos et al. [6].

A solution based on software agents has the potential of providing flexible, controlled (software-only) access to unmodified confidential disaggregate data, and returning only results that do not expose any person-identifiable details. Such a solution is thus appropriate for micro-scale geographical analyses where no person-identifiable details are required in the final results or outputs, i.e., only aggregate results are needed in the final report(s). Furthermore, software agents enable post-coordinated analyses to be designed and carried out on the confidential database(s), as needed, compared to a more conventional solution based on the Web Services model that would only support a rigid, pre-coordinated (pre-determined) and rather limited set of analyses [6].

Geoprivacy has received much attention during the COVID-19 pandemic in the context of digital contact tracing applications and techniques [7, 8]. The privacy-preserving, decentralised ‘Apple-Google Exposure Notification API (Application Programming Interface) using Bluetooth for proximity sensing’ is a notable development in this respect [9]. This method exchanges anonymous user keys or codes, and does not use GPS–Global Positioning System, cellular or WiFi network location.

## Synthetic data generation for privacy preserving sharing of health data

The demand for public health data has increased dramatically in recent times. The COVID-19 pandemic has also been an important driver. Despite that demand, the ability to get access to such datasets has been challenging. Privacy regulations are often being interpreted to limit access to datasets.

De-identification techniques have been developed to address the privacy concerns with sharing health data. However, the increasing number of re-identification attacks has created a negative narrative around de-identification, and reduced trust in de-identified data by regulators and the public.

Synthetic data generation (SDG) is a more recent set of techniques to create non-identifiable datasets. They also are seen as producing better data quality than standard de-identification methods [10]. The purpose of this section is to provide a brief overview of SDG methods. Our focus will be on structured datasets as opposed to, for example, synthetic text or images.

In addition to enabling data sharing in a privacy-preserving manner, SDG has a number of other use cases, such as data augmentation and data amplification [10]. These can be powerful capabilities for health data analysts to deal with small datasets and to accelerate research studies.

### How SDG works

The type of SDG that we will discuss here is where an initial real dataset is used to train a machine learning model. Examples of machine learning models that are often used are Bayesian networks, sequential decision trees, generative adversarial networks, recurrent neural networks, and variational autoencoders. Sometimes these are also combined to work with more complex longitudinal datasets. The trained model is called a ‘generative model’.

Once the generative model is trained, new data can be produced by, for example, sampling from generative model or feeding it new random datasets. Records in this new data do not have a one-to-one mapping to the records in the real dataset because they are generated from a model.

Methods for training generative models on tabular data are quite well developed and work well in practice. Generative models for longitudinal health data remain in the formative stages, although much progress has been made in the last few years. Long sequence datasets, such as data from wearables, remote patient monitoring systems and movement trajectories, require a different type of generative models than short sequence datasets, and these are also a topic of active research.

Geographic information that is represented as postcodes/ZIP codes or counties, for example, can be easily modelled using commonly used generative models for tabular and longitudinal datasets. This type of information would be treated as high cardinality categorical data. Techniques such as target encoding and categorical data embedding layers to efficiently encode that information can be applied in these cases.

Point location information has to be treated differently for optimal results. Treating these as continuous variables may result in implausible points (e.g., on top of mountains or in the middle of the ocean). One approach has been to map these to grids and generate values within each grid. Further considerations are required for trajectory data (e.g., car trips) since the path from source to destination must map to plausible routes.

### Privacy risks in synthetic data

There are three types of privacy risks that are deemed relevant for synthetic datasets: identity disclosure, attribute disclosure and membership disclosure. This assumes that the synthetic data itself will be shared. However, one can also share the generative model with the data users and let them generate synthetic datasets directly (for example, by providing API access to the generative model). There are additional privacy risks that are important to consider for the generative models since adversarial attacks can recover training datasets from machine learning models.

It is commonly believed that because there is no one-to-one mapping between the synthetic records and real records that there are minimal privacy risks for synthetic datasets. However, even though a synthetic record is generated from a model it can still be matched to a real record. This is a form of identity disclosure.

In practice, we consider that an adversary has background knowledge about individuals in the form of quasi-identifiers. These are variables that are in the real and synthetic datasets for which the adversary has correct values about one or more individuals. It is possible for a synthetic record to match a real record on these quasi-identifiers. Even though the synthetic record may not belong to a real person, it may be possible to learn something new about that person if the remainder of the variables (the non-quasi-identifiers or ‘sensitive variables’) are the same or similar between the matched real and synthetic records. This would be an attribute disclosure.

Identity disclosure can occur if the generative model is overfit and synthetic records are replicates of the real dataset. Assuming that that is not the case, then identity disclosure by itself in the context of synthetic data is only problematic if we learn something new about the record that has been matched. Similarly, attribute disclosure in the context of synthetic data is only problematic if we matched the synthetic record with a real record. Therefore, attribute disclosure conditional on identity disclosure is the first form of privacy risk that needs to be managed [11].

Membership disclosure is when an adversary is able to determine that a real person from the same population was in the training (real) dataset. For example, if the real dataset pertained to individuals who participated in a cancer study, then the adversary would know that the target individual has been diagnosed with cancer by being a member of the training dataset. Metrics have been developed to quantify membership disclosure risk [12].

Because privacy metrics have been developed, they can be used during the training of the generative models by including them in the loss function used in hyperparameter tuning, or they can be used for post-hoc evaluations of synthetic data privacy risks. While there is still active research work on improving over the current privacy models, especially for longitudinal datasets, we have reasonable models available today.

A key question is then what is good enough privacy? With quantitative metrics of privacy risks, it is possible to be precise about acceptable privacy risk thresholds. There are many precedents of different organizations around the world that have set these thresholds defining when a dataset is deemed to non-identifiable (see the review in Ref. [11]).

Another approach that has been used to train privacy preserving generative models is differential privacy. However, the evidence that has been emerging suggests that the utility of differentially private synthetic datasets can be low [13, 14], plus appropriate parameterisations for data releases have not been agreed to Ref. [15].

### Evaluating data utility

By maximising synthetic data privacy, we would be diminishing data utility. In general terms, utility is defined as the quality of the synthetic dataset. Therefore, a balance between the privacy and utility is required to ensure that both objectives are met during SDG.

Utility is captured in utility metrics. Utility metrics are useful, for example, in hyperparameter tuning when training generative models, comparing different generative models, and in communicating data quality to the ultimate users of the synthetic datasets.

Firstly, we need to define what synthetic data utility means. There are three dimensions to conceptualising utility.

A synthetic dataset can be used for replication of an already completed analysis using the real dataset. For example, if a journal requires datasets used in its papers to be made available to allow others to replicate the published analysis and results, a synthetic version of that dataset can be shared. High utility is achieved if the conclusions of the published results are the same as the conclusions from the synthetic dataset. Another definition of utility is valid population inference. For example, a synthetic EHR (Electronic Health Record) dataset can be shared to allow researchers to perform any new analyses. The objective of this new analysis is to draw inferences about the population, and therefore utility is defined as the validity of these inferences (such as bias, precision, confidence interval coverage and statistical power).

Another way to think of utility is with respect to either the synthetic dataset or the generative model. A generative model can be used to stochastically produce many instances of datasets. Generative model utility is useful for hyperparameter tuning and comparing different models. A model's utility can be defined as the average utility of all of the datasets that it produces. This is important for model evaluation because any specific dataset has a utility sampled from a model utility distribution and therefore may not reflect the overall performance of the generative model.

Finally, utility metrics can be defined as ‘broad’ or ‘narrow’ [16]. A narrow utility metric reflects a particular analytic workload. For example, the difference in the AUC (Area Under the Curve) for a logistic regression binary prediction model built using the real and synthetic datasets would be a narrow utility metric. In this case the workload is a binary prediction using logistic regression. A broad utility metric is one that does not take into account the specific workloads that the synthetic data will be used for. It is intended to be generic and reflective of how good the synthetic dataset will be across multiple workloads. Typically, these broad utility metrics are defined in terms of the distance between the joint distributions of the real and the synthetic datasets. For example, a multivariate Hellinger distance would be a broad utility metric.

A key criterion that all broad metrics should meet is that they are predictive of narrow utility metrics. If a broad utility metric is not predictive then it is not very useful. The whole objective of having a broad utility metric is that it tells us something about the behaviour of the synthetic dataset(s) on realistic workload(s).

With this general framework we can then define a family of utility metrics that can be used to evaluate generative models and specific synthetic datasets under different circumstances.

Utility can also be extended to cover the structure of the synthetic dataset [17]. For example, if the use case is software testing, then maintaining the structure and format of the original dataset becomes very important. However, here we are limiting ourselves to analytic use cases.

### Common questions about SDG

Some of the more common questions about SDG are addressed below. These are applied questions in that they represent queries from users of this approach for sharing data. The reader should also note that the field is evolving relatively quickly, and therefore over time the responses to these questions will likely change.

Have regulators accepted synthetic data? It is relatively easy to make the argument that SDG is another privacy-enhancing technology for creating non-identifiable data. Therefore, the obligations and benefits of generating non—identifiable datasets and processing non-identifiable datasets would also apply here. A legal analysis of how the GDPR (EU General Data Protection Regulation), CCPA (California Consumer Privacy Act) and HIPAA (Health Insurance Portability and Accountability Act) treat non-identifiable data in general, and synthetic data specifically, has been provided elsewhere [18].

Is it necessary to know how the data will be analysed to synthesise it? As noted above, the general answer is no. One of the benefits of SDG is that the synthetic datasets should be useful for multiple analytic workloads. However, if the specific workload is known a priori then a narrow utility metric can be used as part of the loss function when training the generative model. In such a case, the synthetic dataset will be better calibrated for that particular analytics workload but may not be as useful for a different workload.

How large does the real dataset need to be? Small datasets, such as for rare diseases, are challenging to de-identify because their populations are small which makes patient re-identification risk higher. This makes such datasets attractive inputs for SDG methods. Whether it is possible to train a generative model on a small dataset will depend on the specific modelling method. For example, a gradient boosted decision tree that is used as part of a sequential synthesis method [19] will have different minimal sample size requirements than an artificial neural network with a certain number of nodes, or a Gaussian copula which is used as the generative model which could have even smaller dataset requirements. There is no general answer to this question—it will depend on the machine learning methods being used.

Are there known weaknesses with SDG methods? One of the known challenges with generative models is the ability to model rare events in a larger dataset. This is a challenge in general for model building. A common pragmatic approach to remedy this is to define cohorts where these events are not as rare and use the cohort as the input dataset. However, this is an on-going research topic.

## Privacy-preserving methods should form part of a wider framework

All the above-mentioned privacy and confidentiality-preserving methods are not a substitute for secure and ethical conduct, and a comprehensive health research/public health framework for the appropriate disclosure, use and dissemination of data containing personal identifiable information is required.

The aforementioned methods are a key component of such a framework. Other important ingredients towards such a framework include harmonisation of privacy legislation with clinical/health research and public health requirements, fostering successful partnerships between relevant stakeholder organisations with proper collaboration agreements, bureaucratic simplification, increased multidisciplinary discourse, education (of researchers and data custodians, but also the general public whose personal data are being sought), and development of PET (Privacy Enhancing Technology) toolsets, algorithms and guidelines for using and reporting on disaggregate data [1, 20].

The general public should be able to clearly understand how their data are being used in order to make informed consent and choices. Transparency regarding data uses and 'sunset clauses' (clearly stating when data uses will be complete or cease) are key here [21]. Where possible and applicable, individuals should be given (full or adequate) choice and control over, for example, opting-in/out, granularity and level of data sharing, personal (own) data downloading, consent withdrawal, right to erasure (right to be forgotten), etc.

## Select highlights from the AAG GeoEthics Webinar on ‘ethical issues surrounding the use of geospatial data in health research during the COVID-19 pandemic and beyond’

On 2 December 2021, the AAG and the Institute of Space and Earth Information Science at the Chinese University of Hong Kong jointly organised a webinar to explore the ethical issues surrounding the use of geospatial data to address public health issues during challenging crises, such as the COVID-19 pandemic. The webinar featured speakers and panellists from the UK, US and Hong Kong, including Maged N. Kamel Boulos, Song Gao, Mei-Po Kwan, Ada Lai-ling Chung and Douglas Richardson. The webinar presentations and discussions covered a wide range of topics, including privacy and confidentiality-preserving solutions, addressing people's geoprivacy concerns in times of pandemics and the legal issues involved in using individual-level confidential geospatial data for controlling pandemic spread, and IRB (Institutional Review Board) issues in health research, among other topics.

### Using mobile phone data to understand human mobility patterns and the COVID-19 pandemic

Among the various types of person-specific spatial data, mobile phone data are highly useful for understanding the relationships between human mobility, pandemic control measures, the spread of pandemics, such as COVID-19, and their social implications. For instance, human mobility patterns and social contacts derived from mobile phone data are important indicators for understanding coronavirus transmission, the heterogeneity of human responses and adherence levels to various control measures, thus informing public health decision-making during the COVID-19 pandemic [22–24]. Reduced daily mobility and travel trips may help limit people’s exposure to coronavirus during large in-person gatherings. Gao et al. found the positive associations of state-specific rates of COVID-19 confirmed cases with the change rates of median travel distance and median home dwell time of over 45 million anonymous mobile phone users in the US [25]. Researchers have also used such large-scale mobile phone location tracking data in mobility-augmented epidemic modelling frameworks for reconstructing and predicting the geographic spread of COVID-19 and understanding the health inequities among different socioeconomic groups and geographic neighbourhoods [26–28].

Although anonymous mobile phone and social media location tracking data are very important for monitoring human behaviours in COVID-19 responses (e.g., [29]), they also raise critical issues of geoprivacy and ethical concerns. Users’ identity information and personal sensitive locations, such as home and workplaces, may be disclosed through location data mining and spatial reverse engineering, even after geomasking. As researchers continue to develop and refine their approaches and technical solutions to protect individuals' geoprivacy in health research, they need to strike the right balance between user privacy, data analytical utility and uncertainty [30].

### People's acceptance of location-aware COVID-19 control measures

Kim and Kwan examined people's privacy concerns, perceptions of social benefits and acceptance of various COVID-19 control measures that harness location information using data collected in the US and South Korea. They found that people have higher privacy concerns for methods that use more sensitive and private information. They also observed that people's perceptions of social benefits are low when their privacy concerns are high, indicating a trade-off relationship between privacy concerns and perceived social benefits. Furthermore, the results from their study suggest that people with a stronger collectivist orientation (e.g., South Koreans) tend to have higher acceptance for pandemic control measures because they have lower privacy concerns and perceive greater social benefits associated with the measures [31].

A second study using additional data collected in Hong Kong found that when compared to people in the US and South Korea, people in Hong Kong have a lower acceptance rate for digital contact tracing and higher acceptance rates for self-quarantine monitoring using e-wristbands and location disclosure. Additionally, young people (age < 24) and women in Hong Kong and South Korea have greater privacy concerns than men. Age and gender differences in privacy concerns, perceived social benefits and acceptance of COVID-19 control measures in Hong Kong and South Korea are larger than those in the US [32].

The critical insight obtained from these two studies is that prior experience of pandemics (e.g., SARS—Severe Acute Respiratory Syndrome back in 2003), geographic context and culture (e.g., people's individualist or collectivist orientation) play important roles in shaping people's geoprivacy perceptions and acceptance of different COVID-19 measures. Thus, governments around the world should pay special attention to how the specific history and cultural context of their society affect what pandemic control measures are more acceptable and likely to be effective, and how a reasonable trade-off between public health security (disease control) and geoprivacy protection can be achieved.

### Geoprivacy-preserving pandemic control measures

From a privacy protection point of view, generally, the more sensitive the data are, the more concerns there would be from the public about the collection, use and disclosure of these data. The Office of the Privacy Commissioner for Personal Data in Hong Kong conducted a survey in 2020 for the Global Privacy Assembly, which is an international forum for data protection authorities, on privacy issues arising from pandemic fighting measures. Among the 32 surveyed jurisdictions, only 25% (8 jurisdictions) reportedly incorporated location data or location tracking in their contact tracing measures, while the vast majority (75%), many of which were European countries, decided not to do so out of geoprivacy concerns. Another noteworthy finding in the study was that almost 70% of the jurisdictions surveyed consulted their data protection authorities concerning data protection or privacy impact assessments or other privacy issues related to the development of digital contact tracing measures. New or amended laws were also introduced expeditiously to either address privacy concerns or serve as the legal basis for the collection or use of data. While some jurisdictions, such as Australia and Singapore, implemented statutory restrictions on the use or access to contact tracing data, some others, like Slovakia and Bulgaria, introduced new or amended laws for the processing or collection of personal data. In Bulgaria, for instance, the law was amended to obligate the collection of the location data of persons who violate quarantine orders for contact tracing purposes [33].

As mentioned earlier, transparency is of utmost importance in gaining the trust of stakeholders. Organisations are recommended to spell out, inter alia, the purpose(s) of data collection and the classes of persons to whom data may be transferred at the time or before they collect personal data [34]. It is noteworthy that as a matter of data protection principle, personal data cannot be used for a new purpose other than the original purpose(s) for which the data are collected [35].

In Hong Kong, the general public cares a great deal about their geoprivacy, and the Hong Kong Government has made many efforts to respect and protect geoprivacy in its implementation of measures to fight the pandemic. For example, in an online COVID-19 dashboard which has been set up to disseminate information to the public about the geographic distribution of confirmed cases, no personally identifiable information, such as names of individuals or floor numbers in a multi-story building, are disclosed on the dashboard to protect the geoprivacy of infected persons [36]. In addition, in Hong Kong’s COVID-19 contact tracing app ‘LeaveHomeSafe’, users’ visits to premises are recorded through the scanning of QR codes posted at the entrances to the premises. The app does not have location tracking functions and does not collect users’ GPS data. In other words, the app performs its contact tracing function without tracking users’ movements [37].

The contact tracing app in another Asian jurisdiction, Singapore, also demonstrates how authorities protect individuals' geoprivacy. The Singaporean app uses Bluetooth proximity tracing in smartphones to record close contacts of individuals. While the records can be stored centrally by the government, the app itself does not have location-tracking functions. Notwithstanding that, there was an outcry when it was found that data collected by the app might be accessible by law enforcement agencies. This prompted the Singaporean Government to introduce legislative amendments to restrict police access to the data collected by the app to only seven types of serious crimes [38].

In the UK, the NHS COVID-19 contact tracing app uses the Apple-Google Bluetooth-only method. It is decentralised in that all exchanges among users and between users and the ‘central system’ are in the form of fully anonymous user and diagnosis keys or codes, and the anonymous list of users (anonymous keys) that a user has come in contact with never leaves the user's smartphone [9, 39]. The UK Health Security Agency has been very transparent in its communication with the public about the app, publishing an exemplary detailed guidance about the way the app operates and user data journeys in it [39]. The use of the app is voluntary in the UK; people are free to install it, uninstall it, or not install it at all.

### Codes of ethics, geospatial technologies and geoprivacy in the non-government sectors

In the non-governmental and private sectors, privacy protection needs to be incorporated into professional codes of ethics to ensure the proper protection of people's geoprivacy. For instance, AAG's statement of professional ethics was first put in place in 1998 and has been updated periodically. It lays out several fundamental ethical injunctions, such as the right of informed consent, the requirement to share research results and the need for benefits to the community, all as part of the need to prioritise the “dignity, safety, and well-being” of human subjects, described as “individuals and communities”.

The AAG’s statement of professional ethics is built upon principles widespread among the ethical instruments developed internationally and domestically, particularly with regard to research with human subjects, including biomedical and public health research. With its emphasis upon ‘dignity’, this code of ethics also appeals to standards consistent with human rights. The code states categorically that working with human subjects must include concern for “the basic human rights of affected individuals”, and treats “the role of human rights, social justice, or ethics of care” as equivalent in the overall pursuit of 'well-being'.

The code also gives particular attention to uses of “research involving geospatial technologies”, such as geographic information systems (GIS) and GPS which introduce ‘special challenges’ of an ethical sort, including, but not limited to, questions of privacy, confidentiality, data collection and analysis, community interests and ownership of information. The code of ethics goes on to further specify a variety of potentially problematic applications, depending on context, of some geographic technologies involving automatic tracking of peoples’ locations and movements; uses of images from satellite, aircraft and ground-based sensors; and the use of geographic location, or coordinates, to link personal data.

At the same time, of course, research on, and involving, geospatial technologies can and does generate important scientific advances, as well as significant societal and environmental benefits. Hence, geospatial technologies also contribute to the human right to the benefits of science. Ethical considerations therefore also need to include the opportunities that geospatial technologies provide to catalyse interdisciplinary research, scholarship and teaching and to drive innovation in science, business and society [40]. For example, Geographic Management Systems (GMSs) enable core daily operations management within most governmental and business organisations. GMSs build on the capacity of integrated, real-time and mobile GPS/GIS technologies to create highly interactive real-world, real-time mapping and management environments. They permit the monitoring, modelling and coordination of dynamic spatial activity for day-to-day operations management functions in business, government, international agencies and non-governmental organisations. Currently evolving examples range from simple applications, such as real-time.

management of ambulance or fire vehicle fleets, to more complex activities, such as the continuous, interactive management across space and time of extensive fixed and mobile assets and workforces, e.g., for major electric utility companies, governmental social services or environmental protection agencies, or international disaster and humanitarian relief operations.

The ethical scenarios raised by GIS, GPS and related geospatial technologies include potential conflicts and potential benefits to society. Both must be acknowledged and analysed. Cases of ethical concerns illustrate the special challenges of rapidly evolving new location-aware and location-based technologies and services. Geolocational data, derived from activities such as tracking mobile phones without the consent of users, also raise new questions about the confidentiality of databases with sensitive private information. Researchers and practitioners' creation and use of new geospatial technologies will always introduce many new ethical frontiers with respect to location privacy and the ethical collection, management, distribution and use of geodata. Yet, geographic information science and technologies are also playing essential roles in shaping the future of scientific research in many scientific and medical disciplines. Integral to achieving these benefits of science to society is the ethical responsibility to guard against potential abuses of our powerful new technologies. The Locus Charter aims at improving our "understanding of technology risks, so those can be managed, and the many benefits of geospatial technologies can be realised for individuals and societies’ [41]. More information on GeoEthics is available at Ref. [42].

## Conclusions

This article, conceived as an update and supplement to our original 2009 paper [1], provided a summary of the latest geoprivacy-preserving methods that are in use today in public health interventions (e.g., COVID-19 digital contact tracing) and health research involving disaggregate geographic data about individuals. We also discussed some key location privacy challenges, especially those encountered during public health crises, such as the COVID-19 pandemic. Despite all known challenges, reconciling public health common good and individual privacy concerns is today one step closer, thanks to newer privacy-preserving methods, such as (quasi) synthetic data generated from real data using machine learning and anonymous Bluetooth-only digital contact tracing. However, to fully achieve their goals, privacy-preserving methods should form part of a wider ethical, legal and socio-technical framework for the appropriate disclosure, use and dissemination of data containing personal identifiable information. Interested readers are referred to the original 2009 article [1] for important additional details and insights that are not covered in this 2022 update.

## Data Availability

Data sharing is not applicable to this article, as no datasets were generated or analysed for the current paper.
